# Hospital and Asvlum Construction

**Published:** 1900-12-01

**Authors:** 


### HOSPITAL AND ASYLUM CONSTRUCTION.

### FIRE-RESISTING MATERIALS.

There can, we imagine, be no two opinions as to the supreme importance of taking every possible precaution in the construction of hospitals and asylums to prevent the spread of fire in the event of an outbreak. While there are many systems of so-called fireproof construction, it can scarcely be said of any of them that they are really fire-resisting. The immense destruction of buildings in Paris during the Commune proved to demonstration the danger of trusting to iron and concrete, since the iron, when exposed to great heat did more damage to the structure than if the floors and their supports had been of wood and been rapidly burnt away. While during the last thirty years many and valuable improvements have been made in systems of fire-resisting construction, we are still largely dependent on the use of wood for such things as doors, windows, and floors; and it is, of course, with the use of a soft wood like deal that the element of danger comes in. If, therefore, the wood used in a building can be rendered in any way fire-resisting, a very great step will have been taken toward ensuring the complete protection of a building. The London Non-flammable Wood Company claim by their process to render soft woods, such as deal, pine, &c., not indeed fire-resisting, but non-flammable—that is to say that whatever heat be applied to wood treated by the company's process, it will never ignite in the ordinary sense of the word. It will carbonise or char, but no flame will be produced. This is, of course, an important and valuable invention. Wood is naturally the material which plays the most important part in the spread of fire. If, therefore, the wood as an active agent in the spread of fire can be eliminated, the possibility of confining a fire within comparatively small limits is largely increased.

The process by which the wood is rendered non-flammable is as follows:—

First the wood is subjected to certain changes of temperature in air-tight cylinders in order to free it from its volatile and fermentable elements and to prepare the pores to receive the chemicals which are to render it non-flammable. The chemicals are then forced in under hydraulic pressure until the wood is thoroughly saturated. The wood when saturated is more than double its original weight. It is then re-stacked and run into a drying kiln where by the aid of heating apparatus and fans and a condensing apparatus the moisture is eliminated and the fire proofing ingredients are closely incorporated with the cellulose in the pores of the wood. Besides rendering the wood non-flammable the saturation with antiseptic material prevents the occurrence of decay.

The process was put to a severe test under the direction of the British Fire Prevention Committee in September, 1899. Briefly, the test consisted in subjecting a partition made of wood prepared by the company's process to a heat reaching to about 1300° F. The partition was erected in a hut, the inside of which measured 10 feet and 7 feet 5 inches high. Ample provision was made for the supply of air, and heat was applied by means of gas flames. In the words of the report no "incandescence or spread of flame was observed, nor did the material, as far as could be seen, contribute in any way to the spread of flame; the surface of the boards was charred."

In another and perhaps more striking experiment, conducted by the company's officials, two huts were prepared, one made of non-flammable, the other of ordinary wood. Both huts were subjected to the direct flames of fires from piles of wood saturated with kerosene. In twenty minutes from the start, the untreated hut was a heap of ashes, while the other was intact, and the interior sufficiently cool to allow of its being entered with comfort.

There can, we think, be no question as to the value of the invention, and the only obstacle to its adoption generally is the cost of the process. Doubtless with extended knowledge and improved apparatus, the company will find themselves able materially to reduce their present prices.

